# A Clinical Audit of Surgical Site Infection Surveillance in a Maxillo‐Facial and Oral Surgery Unit in an Academic Hospital Complex in South Africa

**DOI:** 10.1111/iwj.70196

**Published:** 2025-04-27

**Authors:** Emmy Ngoakoana Nokaneng, Samantha L. Holloway

**Affiliations:** ^1^ Maxillo‐Facial and Oral Surgeon University of Pretoria/Steve Biko Hospital Complex Pretoria South Africa; ^2^ School of Medicine Cardiff University Cardiff UK

**Keywords:** infection control processes, oral and maxillofacial surgery, prevalence/incidence, surgical site infection, surveillance programme

## Abstract

The clinical outcomes and financial impact of surgical site infection within South Africa is not well known due to the lack of an established national surveillance programme. The aim of this project was to undertake a baseline clinical audit of surgical site infection prevention in a Maxillo‐facial and Oral Surgery unit using the National Institute for Health and Care Excellence clinical guideline (NG125) as the benchmark standard. The primary objective was to establish a baseline incidence of surgical site infection. This was a prospective and observational clinical audit undertaken at the MFOS unit in a University Hospital in South Africa. Thirty‐seven participants who had surgical procedures were recruited and monitored telephonically post‐discharge for a period of 30 days. The composite compliance rate to the process indicators was 39.86% (95% Confidence Interval 37.25–42.46). The incidence rate of surgical site infection was 14.81% (*n* = 8). The resection of head and neck malignancy contributed majority of the SSI cases (50%, *n* = 4). Five organ/space SSI cases were detected with a mortality rate of 25% (*n* = 2). The higher surgical site infection rates may be associated with the lapses in the infection control practices. For example, the lack of an aseptic technique lack or structured approach to wound management. The main recommendation was the development of evidence‐based surgical site infection preventative strategies that are applicable to the Maxillo‐facial and Oral Surgery procedures to reduce surgical site infection.

**Summary**

- SSI is an infection that occurs after surgery irrespective of the site of the surgical incision(s). The severity of SSIs varies from superficial skin infection to life threatening septicaemia.
- The clinical impact of the SSI was indicated by the high SSI rate relative to previous studies in SA and globally. In this cohort, the SSI resulted in flap failure, sepsis and death. Furthermore, the higher SSI rates may be associated with lapses in the infection control practices.
- SSI surveillance programmes are useful tools in the detection and reduction of SSI.
- It is a necessity to implement preventative strategies such as evidence‐based clinical guidelines and *bundles of care* to ameliorate the impact of SSI.

## 1 Introduction

Surgical site infection (SSI) is an infection that occurs after surgery irrespective of the site of the surgical incision(s) [1]. The severity of SSIs varies from superficial skin infection to life threatening septicaemia [2]. Consequently, they are associated with significant morbidity and mortality of the affected patients with significant number of deaths occurring within 30‐days of the surgery [3].

During Maxillo‐facial and Oral Surgery (MFOS) procedures, the integrity of both the skin and oral mucosa are interrupted with surgical incisions. Inadvertently this predisposes the patient to the risk of local or systemic infection as oral microbes are introduced into the vascular system [4]. Thus, the provision of surgical care within the speciality must be evidence‐based ensuring quality as well as patient safety with best possible outcomes [5]. This should include SSI prevention and quality improvement strategies [6, 7].

Various studies have shown the efficacy of SSI surveillance programmes (SSISP) in reducing the incidence of SSI [8, 9, 10]. The British Association of Oral Maxillofacial Surgeons (BAOMS) have acknowledged the importance of quality improvement strategies within the speciality with the implementation of clinical audits to assess the incidence/ prevalence of SSIs associated with the various surgical procedures [5]. Of significance, the association recognised the importance of clinical audits to understand whether surgical care is delivered in line with standards and afford surgeons to benchmark themselves as well as against other. However, there is a dearth of publications on the efficacy of SSISP with relation to MFOS speciality with the majority of preventative strategies focusing on surgical antimicrobial prophylaxis [11, 12, 13, 14]. Similarly, the prevalence of SSI as well as the impact of SSISP within the MFOS is not well established.

In South Africa (SA), the role of clinical audit in improving patient care is recognised and acknowledged [15]. Thus, its inclusion as a policy directive in the Policy on Quality in Health Care for South Africa, February 2001, which was followed by a clinical audit guideline in 2016 [15, 16]. Accordingly, a clinical audit is a quality improvement process with the primary objective of enhancing patient care and outcomes through a systematic review of standard(s) of care against explicit criteria. Thus, an audit informs clinicians on the standard of care delivered to their patients whilst highlighting lapses in the provision of care, which can then lead to improvements in service delivery and patient outcomes [15, 17].

Therefore, through measuring the prevalence of SSIs within the MFOS unit as well as interrogating the current processes and practices of SSI surveillance, it was possible to appraise and derive fundamental knowledge to inform subsequent preventative strategies such as SSISP including the *bundles of care*. Such “bundles” are implementation tools or evidenced‐based practices that improve the care process and are effective in the prevention of SSI [18, 19, 20, 21].

Thus, the aim of the project was to undertake a baseline clinical audit of SSI prevention in a Maxillo‐Facial and Oral Surgery unit using the National Institute for Health and Care Excellence (NICE) clinical guideline (NG125) as the benchmark standard(s) to audit against [22]. The secondary objective was to establish a baseline incidence of SSI.

## 2 Methods

### 2.1 Setting

This was a prospective and observational clinical audit undertaken at the MFOS unit within the University of Pretoria/Steve Biko Academic Hospital Complex (UP‐SBAHC). This is an 832‐bed public facility with 19 active operating theatres that offers specialised tertiary services to the community of Tshwane in Gauteng Province (Gauteng Province Department of Health, GDoH) [23]. In collaboration with University of Pretoria, the facility is also serves as training platform of trainee clinicians in various disciplines including MFOS.

### 2.2 Criteria and Standards

The criteria and standards, aims and objectives as well as the rationale for choosing the specific criteria and tools for the projects are provided as per revised Standards for Quality Improvement Reporting Excellence (SQUIRE 2.0), which provides a framework for planning, design and execution of healthcare quality improvement systems/projects to facilitate transparency [24] (Table 1).

**TABLE 1:** Criteria and standards.

Measure	Standard/criteria	Indicator
To assess current standards in the prevention of SSI	Baseline assessment tool for SSI: prevention and treatment (NICE clinical guideline NG125) A preselected number of recommendations/criteria (*n* = 30) including recommendations from SA NDoH were assessed	Current activity/evidence Rate of compliance with process measures designed to prevent infection. The rate of compliance was set at 100%
Clinical indicators‐baseline SSI incidence	In‐patient and PDS	SSI rate (The number of SSIs occurring post‐operatively)
To determine lapses in infection control that contribute to SSI	Baseline assessment tool for surgical site infections: prevention and treatment (NICE clinical guideline NG125)	Rate of non‐compliance with process indicators designed to prevent infection.

Abbreviations: NDoH, National Department of Health; NICE, National Institute for Health and Care Excellence; PDS, post‐discharge surveillance; SA, South Africa; SSI, surgical site infection.

The National Department of Health, SA (NDoH) has suggested four bundles of care for SSI, mainly, the appropriate use of surgical antimicrobial prophylaxis (SAP) and hair removal, post‐operative glucose control and normothermia [25]. To assess the current standards in the prevention of SSI, additional process measures were included by the authors, which were deemed relevant to the MFOS unit and procedures. The process indicators were selected to correspond to the peri‐operative phases of the participants' surgical journey, that is, the pre‐operative phase (*n* = 9/30), intra‐operative phase (*n* = 13/30) and the remaining (*n* = 8/30) focused on the post‐operative phase.

The definition and determination of the SSI rate was according to the Centre for Disease Control and Prevention—National Health Safety Network (CDC‐NHSN) [26, 27]. In addition, as MFOS procedures include both an incision of the skin and the oral mucosa, thus degree of wound contamination was classified according to World Health Organisation (WHO) surgical wound classification [28]. Furthermore, the assessment of an SSI considered the various anatomical sites such as the involvement of the bone and the temporomandibular joint (TMJ). Thus, specific definitions applicable to the MFOS procedures were included.

### 2.3 Inclusion and Exclusion Criteria

Inclusion criteria included all patients, adult and paediatric population who had Maxillo‐Facial and Oral surgical procedures under general anaesthetic that involve the incision of the skin or oral mucosa during the clinical audit period with no evidence of infection present or incubating at the time of admission to the MFOS unit [27]. The audit cohort included both ambulatory surgical patients and in‐patients. The audit excluded patients who presented with an infection related to surgical procedures performed outside the audit period; all patients who presented with infection without prior history of surgical procedure as well as all patients who presented with infection with prior history of surgical procedure who were not operated within the unit.

### 2.4 Audit Sample

Consecutively sampling of all patients who had either an elective or an emergency MFO surgical procedure during the period of 01 November 2022 and 31 December 2022 was conducted. Thirty‐seven participants were recruited and were followed up until 31 January 2023 as per CDC‐NHSN recommendation [27].

### 2.5 Data Collection

The baseline assessment tool for SSI (NICE clinical guideline NG125) was utilised to capture the compliance with the process measures [22]. A total of (*n* = 30) preselected process indicators deemed appropriate by the authors for the MFOS speciality were assessed (Data [Link], [Link]). Data was electronically collected on a Microsoft Excel spreadsheet for each patient having a procedure.

The surveillance was *patient‐based* with data collected at an individual level on all eligible participants, with active follow‐up to identify those who developed an SSI [29]. For in‐patients and ambulatory patients, data was collected on the same day as the procedure. The WHO SSI peri‐operative data form was used [28] (Data [Link], [Link]). For the post‐discharge surveillance (PDS), the WHO PDS tool kit was utilised [28, 30] (Data [Link], [Link]). Participants were followed‐up telephonically for a period of 30 days. On each review, three (3) attempts were be made at different occasions before being reported as lost to telephonic follow‐up surveillance [28].

To improve the internal validity of the data collection tools in this setting, a pilot study, which included piloting the data collection tools on 10 patients, was conducted [18, 31]. Based on the experience, there were no modifications made to the methodology and the pilot study indicated that the clinical audit was feasible within the MFOS unit.

### 2.6 Ethical Considerations

Ethical approval was obtained sought from Cardiff University's School of Medicine Research and Ethics Committee as well as endorsement from University of Pretoria's Faculty of Health Science Research and Ethics Committee (SA). The ethics approval reference number is 416/2022. Informed consent for data collection was obtained from the participants.

### 2.7 Data Analysis

Process indicators were calculated as percentages with the number of relevant recommendations per participant as the denominator and the number of recommendations met per participant was the numerator [22]. The process indicators were calculated with a confidence interval (CI) set at 95%.

Incidence rate of SSI was defined as the number of new SSI infections that occurred during a specific period in a defined population [29, 32]. This was reported as the number of SSI per 100 operations [29, 33]. Continuous variables were reported as means as well as standard deviation and analysed using an independent *t* test. Categorical variables such as gender, type of procedure and risk factors were presented in number as well as percent. An odds ratio (OR) was used to identify risk factors associated with SSI and *p* value of less than 0.05 was considered as statistically significant. All statistical analyses were performed using SPSS software (Version 28.0; SPSS Inc., Chicago, IL, USA).

## 3 Results

### 3.1 The Process Indicators

**FIGURE 1:**
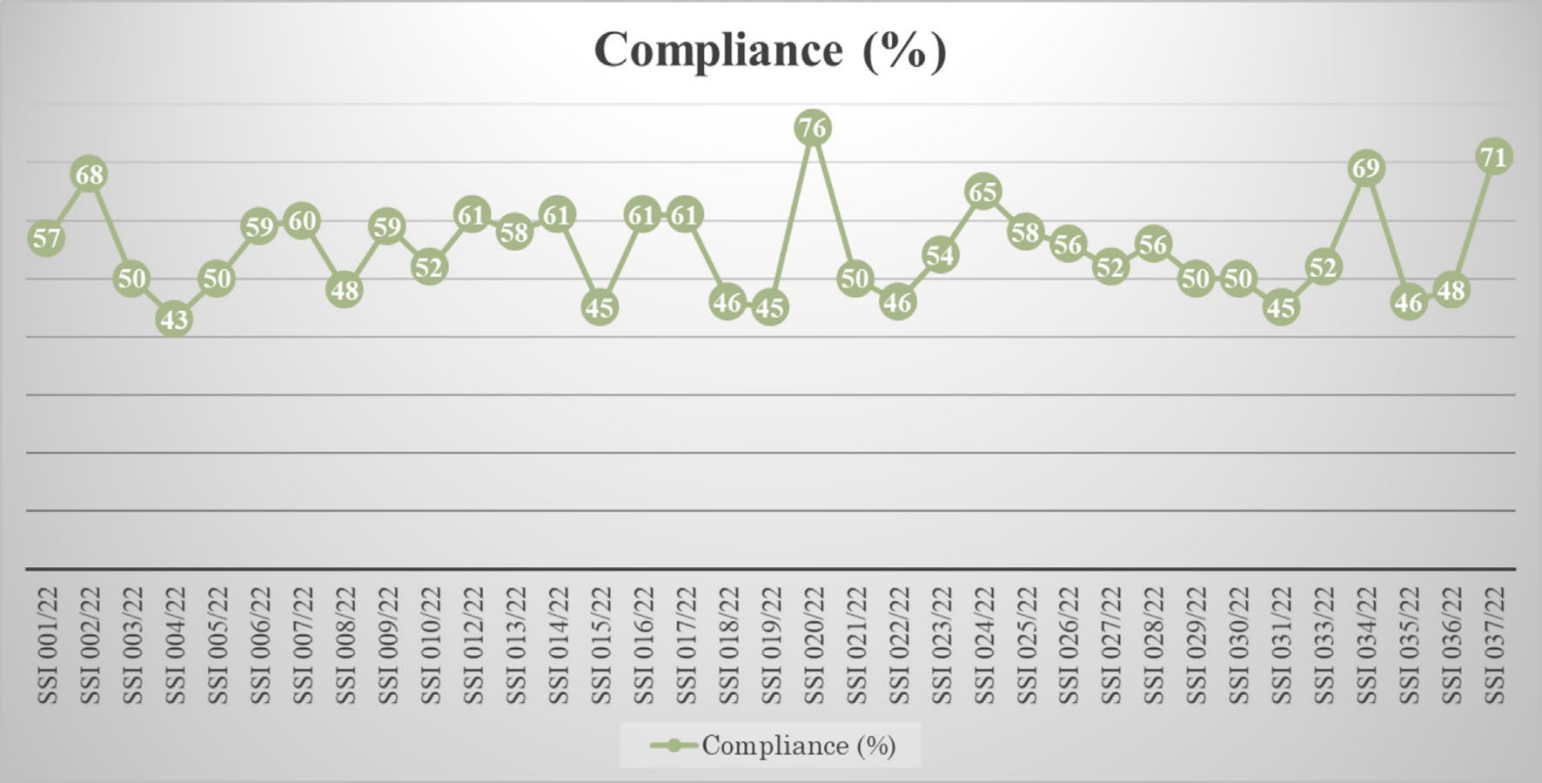
Compliance rate with the process indicators.

The composite compliance rate for all participants was 51.9% (95% CI, 52.3%–57.9%). The minimum and maximum compliance rate were 42.8% and 76.0%, respectively with no outliers. The interquartile range was 12.8%. The median of the compliance rate was 54.5% and mean of 55.1% thus suggesting a tendency towards a right skewed distribution of the data set (skewness = 0.632). Figure 1 shows the trends in compliance rate for each participant. Of significance from the chart is the variability in compliance although none of the participants achieved the targeted adequate compliance rate of 100%.

Process indicators within these phases were also analysed to determine the lapses in infection control within each phase.

### 3.2 The Pre‐Operative Phase

Of the nine process indicators audited, two recommendations were met for all participants in whom the recommendations were applicable. These included not utilising hair removal routinely to reduce the risk of SSI and the removal of hand jewellery before operations. Two recommendations fell short of full compliance, that is, the removal of artificial nails and nail polish before operations and the provision of antibiotic prophylaxis before clean, clean‐contaminated and contaminated wounds and the antibiotic prophylaxis before 120 min preceding surgical incision (97.1%, *n* = 34/35; 97.1%, *n* = 34/35, respectively).

**FIGURE 2:**
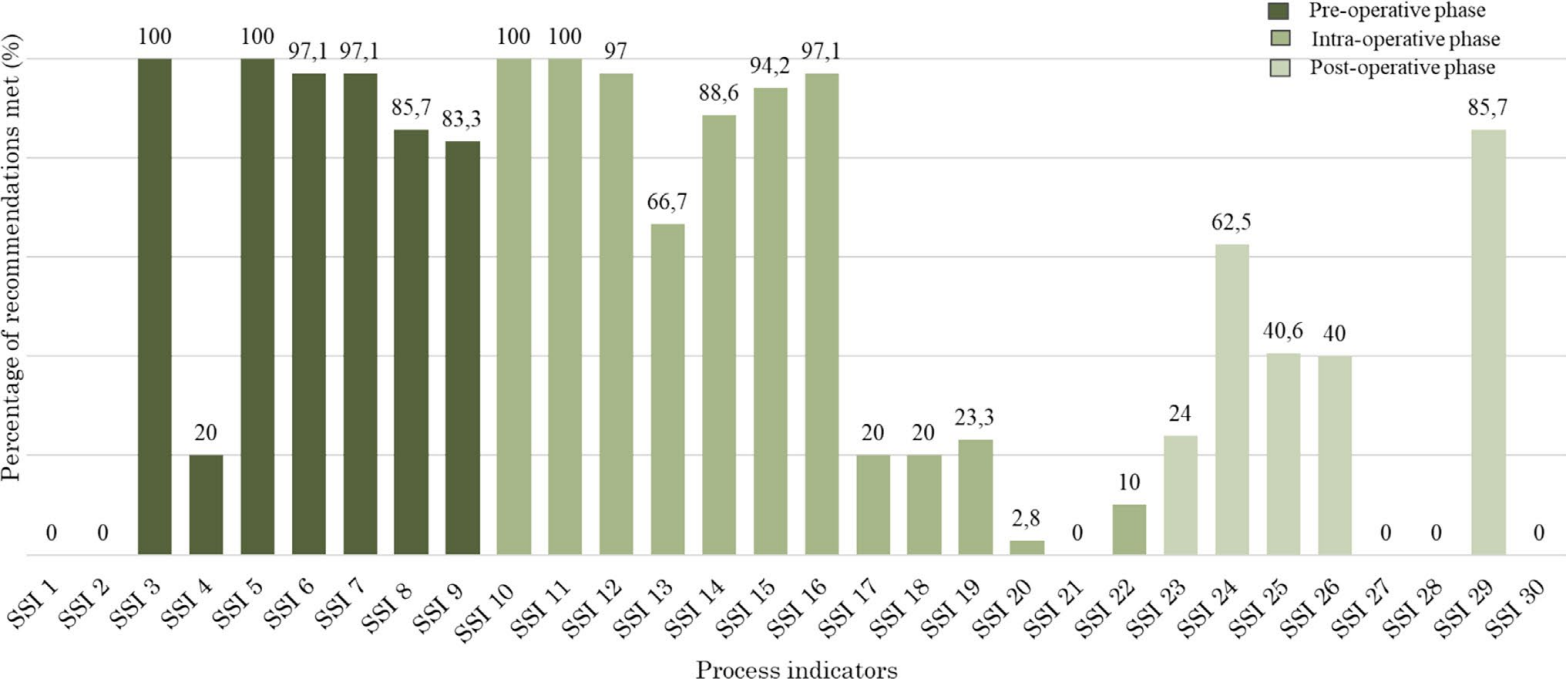
Percentage of process indicator met relative to the participants.

The administration of surgical antibiotic prophylaxis in the 120 min preceding surgical incision was met in 85.7% (*n* = 30/35) whereas the administration of antimicrobial treatment (in addition to prophylaxis) for patients having surgery on a dirty or infected wound was met in just 83.3% (*n* = 5/6) participants. Figure 2 provides an overview on the recommendations met during the clinical audit.

Compliance with the remaining three process indicators were poorly met in participants in whom the recommendations were applicable. These included the advice to patients to have a pre‐operative bath or shower (0%, *n* = 0/35) as well as the use mupirocin in combination with a chlorhexidine body wash before procedures where there is a risk of *Staphylococcus aureus* causing SSI (0%, *n* = 0/35). The recommendation not to use razors for hair removal achieved a 20% (*n* = 2/10) of the participants in whom the recommendation was applicable.

### 3.3 The Intra‐Operative Phase

In this phase, two recommendations were met in all participants in whom the recommendations were applicable with 100% compliance (*n* = 35) in both the pre‐operative handwashing by the operating team prior to the first operation using an aqueous antiseptic surgical solution and a brush as well as the hand washing before subsequent operations. The preparation of the skin at the surgical site immediately before incision using an antiseptic preparation was performed in 97% (*n* = 33/35) whilst the maintenance of normal body temperature (normothermia) (> 36°C) was also met in 94.2% (*n* = 33/35) of the participants. The peri‐operative maintenance of patient's core temperature using warming procedures was achieved in 97.1% (*n* = 34/35) of the participants.

Six of the process indicators were poorly met. These included the use antimicrobial triclosan coated sutures (0%, *n* = 0/35). Similarly, the utilisation of aqueous povidone iodine (PVP‐I) solution for irrigation of incisional wound before closure for the purpose of preventing SSI and the coverage of incisional wound with appropriate interactive dressing were only met in 2.8% (*n* = 1/35) and 10% (*n* = 2/20) respectively.

### 3.4 The Post‐Operative Phase

Overall, there was poor compliance with the process indicators in the post‐operative phase. The highest percentage for compliance were associated with the prescription an antibiotic that covers the likely causative organisms in patients with suspected SSI (85.7%; *n* = 6/7). The non‐utilisation of topical antimicrobial agents for surgical wounds that are healing by primary intention was achieved in 62.5% (*n* = 15/24).

The remaining six process indicators were poorly met. Regarding the recommendations for the overall surgical wound care and management, there was a complete lack of compliance with the structured approach to wound care to improve overall management of surgical wounds including the utilisation of an aseptic non‐touch technique for changing or removing surgical wound dressings was performed as well as seeking advice or assistance from a tissue viability nurse (or another healthcare professional with tissue viability expertise) on the appropriate dressings for the management of surgical wounds that are healing by secondary intention.

### 3.5 Incidence of Surgical Site Infection

Thirty‐seven participants who met the inclusion criteria were prospectively monitored for SSI during the surveillance period. Two participants were lost to follow‐up. Eight cases of SSI were detected giving an incidence rate of 14.81%. The mean age of the participants was 32.36 years (SD 19.03) with a male gender predominance (62.9%, *n* = 22) (Table 2). Regarding the participants co‐morbidities, two of the participants had more than one co‐morbidity, that is, diabetes mellitus (DM) and myocardial infarction in the one participant whilst the second participant had hypertension and previous malignancy.

**TABLE 2:** Demographic data of the pilot and main clinical audit cohort.

Variables		*n* Total *n* = 35	%	Mean	SD
Age	Range	0.7–77		32.36	19.03
Gender	Female	13	37.1		
	Male	22	62.9		
Comorbidities	None	30	85.7		
	HPT	3	8.6		
	DM	2	5.7		
	MI	1	2.8		
	Previous malignancy	1	2.8		

Abbreviations: DM, diabetes mellitus; HPT, hypertension; MI, myocardial infarction; SD, standard deviation.

The majority of the surgical procedures were categorised as semi‐elective (54.3%, *n* = 19) with an American Society of Anaesthesiologist (ASA) classification I (85.7%, *n* = 30). The majority of these procedures were associated with clean‐contaminated wounds (48.14%, *n* = 26) (Table 3).

**TABLE 3:** Surgical demographic data.

Variables		*n*	%	Median	SD
ASA classification		Total *n* = 35			
	ASA I	30	85.7		
	ASA II	4	11.4		
	ASA III	1	2.9		
	ASA IV	0	0		
	ASA V	0	0		
Wound classification		Total *n* = 54			
	Clean	9	16.98		
	Clean‐contaminated	26	48.14		
	Contaminated	15	27.77		
	Dirty/infected	4	7.40		
Urgency of operation	Emergency	0	0		
	Urgent	8	22.85		
	Semi‐elective	19	54.28		
	Elective	8	22.85		
Length of Hospital Stay	Minimum days	0		2.00	8.27
	Maximum days	32			

Abbreviations: 0, ambulatory setting; ASA, American Society of Anaesthesiologist; SD, standard deviation.

**FIGURE 3:**
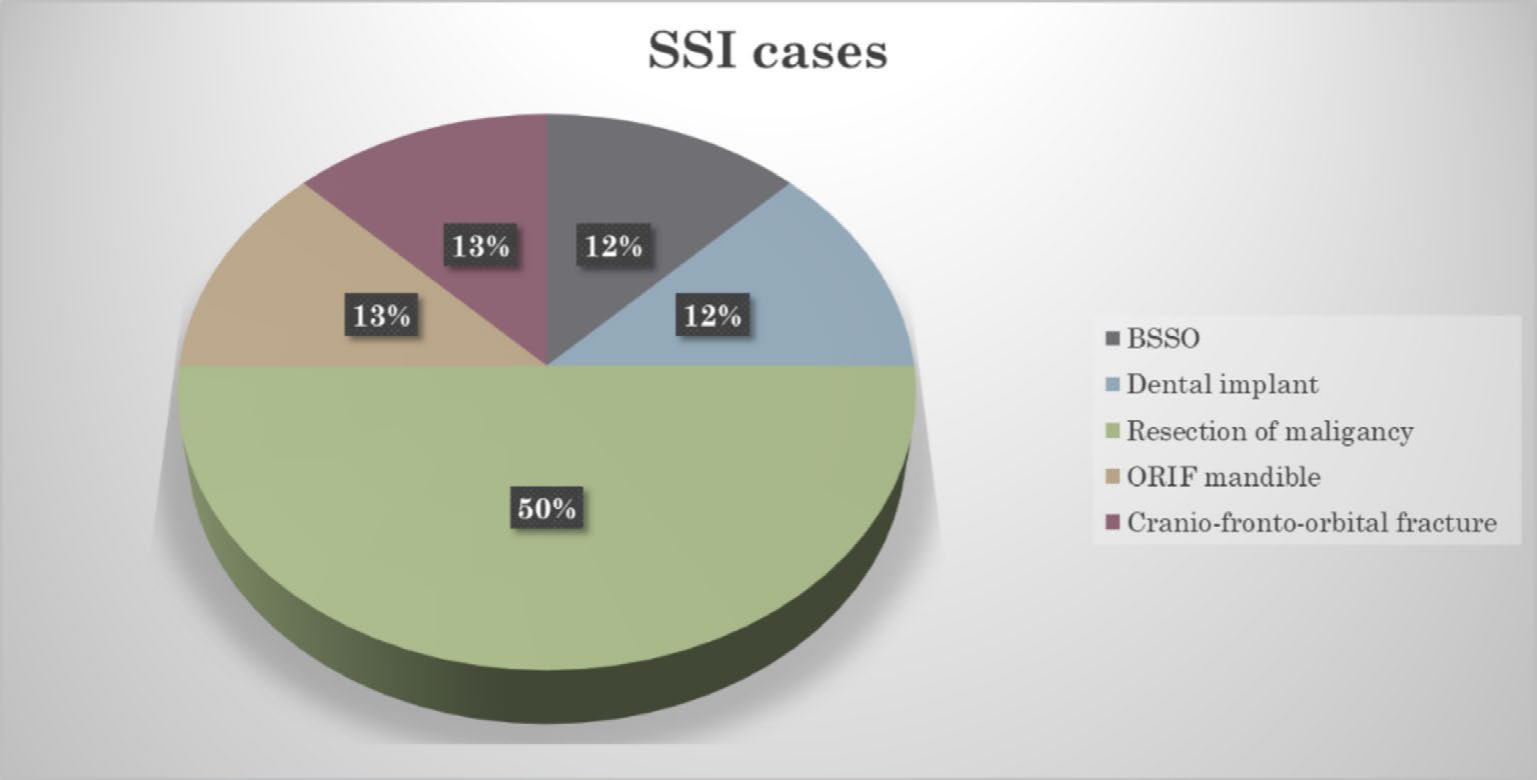
Distribution of SSI cases according to the type of procedures. BSSO, bilateral sagittal split osteotomy; ORIF, open reduction & internal fixation.

**FIGURE 4:**
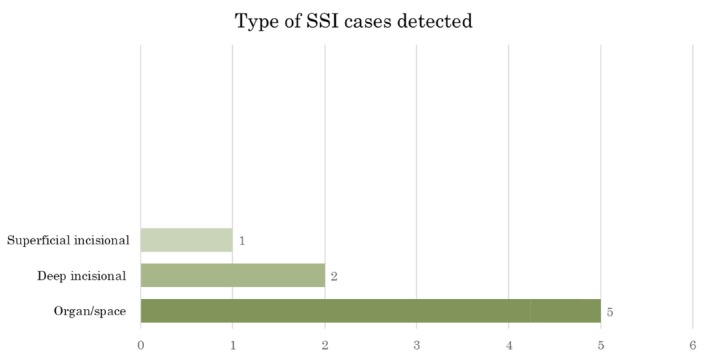
Type of SSI cases detected.

The bilateral sagittal split osteotomy was the most common procedure performed (18%, *n* = 10) followed by an open reduction and internal fixation of mandibular fractures (16%, *n* = 9). However, the resection of head and neck malignancy contributed majority of the SSI cases (50%, *n* = 4/8) (Figure 3). Five organ/space SSI cases were detected (Figure 4).

An SSI was commonly found in contaminated wounds (61.53%, *n* = 8) and in semi‐elective procedures (62.5%, *n* = 5). Six cases (75%) were detected with the PDS an average of 22 days (range 7–51 days) to detection.

### 3.6 Risk Factors Associated With Surgical Site Infections

Males were 4.96 times more likely to develop an SSI than females. An ASA II and ASA III classification was also associated with increased risk of an SSI (OR 5.6). Participants with clean‐contaminated and contaminated wounds were 26.5 times likely to develop an SSI. However, these variables were not statistically significant with *p* values of (0.999; 0.999; 1) respectively (Table 4).

**TABLE 4:** Logistic regression for risk factors associated surgical site infections.

Factor	Odds ratio	*p*	CI
Gender			
Male^a^	4.96	0.999	0.54–23.31
ASA classification			
ASA I^b^	5.6	0.999	0.0
Wound classification^c^			
Clean‐contaminated	26	1	0.0
Contaminated			
No of wounds^d^	4.06	0.304	−1.46—2.45
Urgency of procedure^e^			
Semi‐elective urgent	0.49	1	0.0

^a^ Reference variable: female.

^b^ Reference variable: ASA I.

^c^ Reference variable: clean wounds.

^d^ Reference variable: more than one wound.

^e^ Reference variable: elective procedures.

### 3.7 The Management and Clinical Outcomes of the SSI Cases

Table 5 shows the management of participants with an SSI and the outcomes thereof. The mortality rate was 25% (*n* = 2).

**TABLE 5:** Management and outcomes of participants with a surgical site infection.

Participant code	Type of SSI detected	Management	Outcomes
SSI 001/22	Deep SSI	I&D antibiotics	Resolution
SSI 007/22	Organ/space SSI	Debridement & antibiotics	Resolution
SSI 008/22	Organ/space SSI	RHT antibiotics	Resolution
SSI 016/22	Organ/space SSI	Debridement & antibiotics	Flap failure & resolution of infection
SSI 020/22	Deep SSI	I&D Antibiotics	Resolution
SSI 027/22	Superficial SSI	Antibiotics	Resolution
SSI 029/22	Organ/space SSI	Antibiotics	Death
SSI 037/22	Organ/space SSI	Antibiotics	Sepsis and death

Abbreviations: I&D, incision and drainage; RHT, refusal hospital treatment.

### 3.8 Microbiology Associated With the Surgical Site Infection

Microbial culture was performed in five of the eight of the SSI cases. However, organisms were identified in two of the participants whereas the results of the remaining three specimens could not be traced on the database suggesting that the specimens were not received by the local microbiology laboratory. The isolated organisms included *Staphylococcus aureus* , *Streptococcus spp*. and *Corynebacterium spp*.

## 4 Discussion

In this audit, the composite compliance rate for all participants was almost 52%, which was much lower than expected. On the other hand, there was variability in the compliance rate (43%–76%) in all 35 participants. The compliance rate was calculated utilising those recommendations applicable to the individual participant.

## 5 The Process Indicators

### 5.1 The Pre‐Operative Phase

In the pre‐operative phase, the compliance rate for removal of hand jewellery before operations was 100% and slightly lower for the removal of artificial nails and nail polish before operations (97%). The results are encouraging as this practice is aligned with the NDoH, the WHO as well as the International Wound Infection Institute's (IWII) hand hygiene guidelines [25, 34, 35]. Hand hygiene has been prioritised in the infection prevention programme within public health facilities NDoH [25, 33]. The author observed that hand hygiene posters were displayed above the hand wash basins in every consulting room and scrubbing areas in operating theatres as per guideline NDoH [25, 33]. Flodgren et al. suggested that the proactive dissemination of educational materials through various educational approaches enhances awareness and may be an effective tool in the prevention of SSI and therefore may have enhanced hand hygiene in the current clinical audit [36]. The reason for the lack of compliance with regards to the removal of artificial nails in one participant is unknown but may be due to lack of knowledge by the clinician on the risk of SSI associated with artificial nails. Thus, feedback of the clinical audit results to the clinicians in this regard is important and will serve not only as an opportunity to educate the clinicians but also improve and enhance patient safety going forward [37].

Compliance with antibiotic prophylaxis before clean, clean‐contaminated and contaminated wounds including antibiotic prophylaxis before 120 min preceding surgical incision was 97%. This is contrast to the clinical audit conducted by Brink et al. that assessed the timing and administration of SAP in surgical patients [38]. The SAP was administered in 81.2% (95% CI 78.5–83.8) of the participants and within 60 min to 34.7% (95% CI 31.7–37.7) [37]. There was also a significant difference between the rate of compliance in administration of SAP in the current audit and that conducted in a French hospital network wherein the compliance rate was 64% whilst the administration of SAP within 60 min before incision was 77.6% [39].

The NDoH has adopted the Best Care Always *bundle of care* for SSI as an SSI preventative strategy, which includes the appropriate use of prophylactic antibiotics (including appropriate selection, timing and duration/discontinuation) [25, 40]. In this regard, this may have influenced the current compliance. It is also important note that this was the first clinical audit in the unit and there is no previous data to draw comparisons with. Therefore, the findings of this audit will act as a baseline for future clinical audits in the unit and in SA.

With regards to removal of hair, the compliance rate was high (100%). This was in all the participants in whom the hair removal was required (*n* = 10/35). The NDoH guideline and Best Care Always *bundle of care* includes appropriate hair removal with avoidance of shaving using razors [25, 40]. Thus, it is expected that there would be compliance. In this regard, the compliance rate was poor as razors were used for hair removal (20%). This may also be reflection of the surgeon's lack of awareness of the risk of SSI associated with the use of razors for hair removal. Although, these guidelines have been in place for many years, the barriers to implementation to the guidelines will have to be identified to improve the competence as well as translation of evidence into clinical practice. On the other hand, the disposable single use clippers may be expensive to procure in a low‐ and middle‐income counties (LMICs), which is an important consideration as the audit was undertaken in LMIC wherein resourcing of medical supplies is suboptimal [34]. Although, SA is regarded as a middle‐income country, over the years there has been deterioration in the delivery of healthcare services due to lack of resources [41, 42].

### 5.2 The Intra‐Operative Phase

The preparation of the skin at the surgical site immediately before incision using an antiseptic preparation was performed in almost 97% of cases. In the cases that did not meet the required recommendation, 0.9% saline solution was used for skin preparation. In other cases, the surgical team used either aqueous‐ or alcohol‐based chlorhexidine solution as well as aqueous‐based povidone‐iodine solution, which is aligned with the NDoH and IWII guidelines on antiseptics [25, 35]. A systematic review and meta‐analysis on the efficacy of aqueous solutions in reducing the risk of SSI showed that alcohol‐based antiseptics were more effective than aqueous solutions [42]. In contrast, the more recent findings by NIHR Global Research Health Unit on Global Surgery suggested that there was no significant SSI risk reduction with alcohol‐based 2% chlorhexidine in comparison to povidone‐iodine independent of the surgical wound contamination (clean‐contaminated stratum) 15·3% [223/1455] versus 15·7% [231/1468], relative risk 0·97 [95% CI 0·82–1·14]; contaminated or dirty stratum 28·3% [338/1194] versus 31·8% [371/1167], relative risk 0·91 [95% CI 0·81–1·02] [43]. This may suggest that the NDoH guidelines should be reviewed so that they are aligned with the current evidence.

The maintenance of normal body temperature (normothermia) (> 36°C) was also met in almost all of the participants (*n* = 33). Normothermia is also included in the BCA *bundle of care* for SSI prevention albeit it is mainly recommended for patients having colorectal surgery [25, 40]. In most instances, normothermia was achieved through a forced‐air blanket (Bair Hugger Model 505, Arizant Healthcare USA). This positive outcome will be shared with clinicians to reinforce this in clinical practice.

Overall, six of the process indicators were poorly met. For example, the recommendation to utilise aqueous povidone‐iodine (PVP‐I) solution for irrigation of incisional wounds before closure for the purpose of preventing SSI and the coverage of incisional wounds with appropriate interactive dressing were poorly met. The results of this audit identified that 0.9% saline solution was the most commonly used solution for irrigation of incisional wounds.

The low compliance rate (10%) in relation to the use of interactive dressings is likely to be explained by the inadequate use of interactive dressings as well lack of knowledge by the clinicians on the appropriate dressing to utilise on surgical wounds. However, it should be borne in mind that there is limited availability of interactive dressings within the institution. Therefore, there is a wider consideration regarding the use of interactive dressings on surgical wounds in relation to reducing the risk of SSI. The audit also showed that the surgeons had a preference of wound closure strips (Steri‐strips3 M) for coverage of the incisional skin wounds. Wound closure strips are used to reinforce the tensile strength of those wounds healing by primary intention to reduce the risk of wound dehiscence [44]. Thus, wound closure strips do not have the properties that create a moist, conducive environment that facilitates wound healing [45]. In this regard, they are not recommended in clinical practice. To the contrary, a Cochrane review concluded that there was no evidence to the effect that one dressing was superior to the other at reducing the risk of an SSI nor that covering wounds with any dressing reduced the risk of an SSI [46]. Therefore, in clinical practice the decision of the type of dressing may be left to the clinicians' preference.

### 5.3 The Post‐Operative Phase

In comparison with the pre‐operative and intra‐operative phases of surgery, poor compliance with the process indicators within post‐operative phase was very evident. The recommendation not to use topical antimicrobial agents for surgical wounds healing by primary intention was met in less than 65% of the participants in whom the recommendation was applicable whilst just over two‐fifths did not have prolonged administration of SAP after completion of the operation for the purpose of preventing or to reduce the risk of an SSI.

Currently, the NDoH guideline does not include guidance on these two recommendations [25]. Similarly, there is lack of SAP guideline within the MFOS unit thus the choice of an antimicrobial and duration thereof is left to the surgeon's discretion. This is in contrast to the IWII position that recommends formulation of institutional guidelines on antimicrobial stewardship to guide clinicians on the management of wound infection [35]. Nevertheless, the shortest effective duration of antibiotic prophylaxis for preventing an SSI has not yet been established [47]. However, prolonged antimicrobial administration is not recommended as the patient may be at risk of antimicrobial resistance as well as adverse events [48, 49, 50]. Brink et al. showed that the administration of SAP for 24 h in clean and clean‐contaminated wounds significantly reduced the SSI rate from 19.7% to a mean rate of 1.97 (95% CI 1.79–2.15) (*p* = 0.0029). Findings from the current audit identified that four of the seven patients received prolonged SAP (> 24 h) developed an SSI [37]. A finding that supports the view that prolonged SAP does not offer any additional benefit in preventing SSI [20]. On balance, the existing evidence and the results from the audit indicate that in clinical practice, prolonged SAP may not reduce the risk of an SSI.

With regards to post‐operative wound care and management, the results showed that there was a complete lack of a structured approach to wound care to improve overall management of surgical wounds. Similarly, advice on the appropriate dressings for the management of surgical wounds that are healing by secondary intention was absent. SSI develop primarily as result of wound contamination through microbial invasion as the integrity of the mucosa/skin is compromised by a surgical incision [35, 51]. Thus, it is imperative that post‐operative wound management is integral to the post‐operative care of the surgical patient. The current *bundle of care* and IPC guidelines do not provide any guidance to that effect and currently there is lack of wound management guidelines within the MFOS unit [25]. Thus, recommendations will be made to facilitate the development of evidence‐based wound care and management clinical guidelines.

## 6 The Clinical Indicators

### 6.1 Incidence of Surgical Site Infection

Eight cases of SSI were detected giving an incidence of SSI of 15%. In comparison to the reported incidences of SSI in SA, the current incidence of SSI is significantly higher that the majority of the reported SSI in SA (range 0.65%–13.3%) [52, 53, 54, 55, 56, 57, 58]. Conversely, the current SSI rate was much lower than the SSI rate of 48% reported by Bokop‐Fotso et al. [59].

Inter‐institutional benchmarking is dependent on the standardisation and validation of surveillance systems/programs in place [29, 60]. For example, it is not appropriate to compare health facilities that undertake PDS with those that do not [21].

It is also interesting that the current findings corroborate previous findings that suggest that LMICs have higher SSI rates than high‐income countries. GlobalSurg Collaborative concluded from an international, prospective and multicentre cohort study (which included cohorts from high‐, middle‐ and low‐income countries), that the low‐income countries bear higher burden of SSI related to gastrointestinal surgery than in middle‐ and high‐income countries [3]. The unadjusted incidence of SSI in low‐income countries was 23.2% (*n* = 298/1282) in comparison to 14.0% (*n* = 549/3918) and 9.4% (*n* = 691/7339) from the middle‐ and high‐income countries respectively (*p* < 0.001). Thus, from a global perspective and in comparison, with high‐income countries, the SSI rate is significantly higher with incidences of 1.9% in USA, 5.4% in Switzerland, 2.6% in Italy, 0.4%–8.8% England [28].

### 6.2 Incidence of Surgical Site Infection and Associated Risk Factors

In this clinical audit, a logistic regression indicated that various risk factors such as gender, ASA classification and wound contamination were associated with the development of SSI. However, these variables were not statistically significant with *p* values of 0.999; 0.999; 1, respectively.

It was shown that participants with an ASA II and ASA III classification were also associated with increased risk of an SSI (OR 5.6). Three of the participants who developed an SSI had an ASA II whilst another had an ASA III. Furthermore, two of the participants with an SSI had DM. DM has been suggested as risk factor for an SSI and this may be associated with the increased accumulation of advanced glycation end‐products, which impair wound healing [35, 61].

With regards to type of wound contamination, participants with clean‐contaminated and contaminated wounds were 26.5 times more likely to develop an SSI. Wound categories are regarded as indicator for the risk of SSI [27, 34, 35]. In this clinical audit the majority of the participants with an SSI had contaminated and dirty wounds (75%, *n* = 6/8). In this regard, it is not surprising that the patients developed an SSI.

Taken together, it is important that the pre‐operative assessment of the patients should take into consideration factors that may influence the post‐operative outcomes and every effort should be taken to ameliorate these factors [35]. In addition, the authors recommends a development and adoption of an evidence‐based pre‐operative SSI risk assessment algorithm for clinical practice in the MFOS unit.

### 6.3 The Nature of Association Between the Process Indicators and the Surgical Site Infection Outcomes

The higher SSI rates may be associated with the lapses in the infection control practices (ICP) that have been identified and previously discussed. For example, the lack of an aseptic technique or non‐touch technique in wound care including the lack of structured approach to wound management may have contributed to the development of an SSI in some participants. The IWII recommends the aseptic technique as a framework to prevent wound infection [35]. Thus, it is imperative that an evidence‐based wound management guideline is formulated for the MFOS unit to facilitate a structured approach to surgical wounds. In addition, the establishment a surveillance programme within the unit will facilitate the prompt identification of patient at risk as well as those who develop an SSI within the institution and post‐discharge from the health facility. The findings in this clinical audit have indicated that PDS is a useful tool in the identification of an SSI as 75% of the cases were detected through PDS. This corroborates previous findings that the majority of patients (12%–84%) with SSI present with the infection post‐discharge from hospital [3, 62, 63, 64].

The development of an SSI may also be associated with the lack of compliance with regards to pre‐operative skin preparation. The use of 0.9% saline for skin preparation was probably inadequate to reduce the microbial load especially in patients with contaminated and dirty wounds. Furthermore, the microbial load could have been reduced through the pre‐operative bathing.

Pre‐operative skin preparation is a prescribed standard with the aim of reducing the microbial load on the patient's skin as much as possible before the interruption of the skin barrier with an incision [42]. Alverdy et al. posits that the pathogen(s) may also originate from a distant site within the host such as the oral cavity [65]. A view that supports the application of oral hygiene practices before major surgery to reduce post‐operative complications [66]. The importance of oral hygiene practice before surgical procedures can further be gleaned from the reduction in SSI in response to SAP in patients who practice pre‐operative oral hygiene [67]. This is an important aspect in the surgical care of MFOS patients wherein both the oral mucosa and skin's integrity are interrupted with a surgical incision that inadvertently raises the risk of local or systemic infection as oral microbes are introduced into the vascular system [3, 68, 69]. Thus, the recommendation that in addition to SSI risk assessment, the pre‐operative oral hygiene assessment and oral hygiene programme be implemented for surgical patients.

## 7 Evaluation of the Clinical Audit

### 7.1 The Strengths of the Clinical Audit

The clinical audit was planned, designed and conducted using established frameworks, standards and criteria [22, 27, 28]. The reporting of the results as well as the identification of the strengths and limitations of the clinical audit including the recommendations made for clinical practice were guided by SQUIRE 2.0 [24]. The primary aim of the clinical audit was achieved, that is, to interrogate current processes and practices that facilitated the identification of lapses in infection control practices as well as establish a baseline SSI incidence for the MFOS unit. Therefore, the information derived from the clinical audit will afford the MFOS unit an opportunity to appraise and derive fundamental knowledge that will inform clinicians within the unit on the standard of care delivered to their patients whilst highlighting lapses in the provision of care [15]. Accordingly, these will inform preventative strategies and improve in service delivery and patient outcomes [17].

This is the first quality improvement project as well as the first to establish SSI incidence in SA related to MFOS procedures and thus may be a benchmark for future research on the epidemiology of SSI within the speciality or comparison with other specialities. Furthermore, the possible risk factors were identified and thus may inform the MFOS unit to be proactive in the pre‐operative identification of patients at risk as well as develop strategies to mitigate the development of an SSI in such patients.

The audit also utilised validated tools for the data collection and thus credible data was derived [32]. Although there is no consensus on the gold standard for validation of an SSISP; validation of surgical site infection surveillance (SSIS) data is crucial to ensure its scientific credibility as well as to enhance identification of methodological challenges within the surveillance programme such as potential sources of selection and detection bias [70, 71].

### 7.2 Limitations of the Clinical Audit

Active surveillance has high sensitivity and specificity; however, it is a resource‐ and time‐consuming activity and therefore may not be feasible in the settings with limited resources [72, 73]. The authors had a similar experience with the current project wherein there were conflicting responsibilities between the project and work. This supports the view that for a successful SSISP, there should be an organisational culture that promotes and enhances implementation of surveillance programmes and data collection as well as adequate human resources including personnel dedicated to data collection [73]. In essence, the success of the SSISP within the MFOS unit will be dependent on the collaborative effort of the personnel within the unit and the institution.

A further limitation with the current project was the small sample size in comparison to other studies in SA and globally. However, as this was a clinical audit a small sample is considered adequate as the process evaluates whether practice complies with standards [17]. Similarly, the NDoH framework stipulates that *only the minimum data required by the objectives of the audit* should dictate the sample size [15]. A sample size of 30–50 cases is recommended for clinical audits based on the rationale that if care is not provided according to the set standard(s) in a cohort of 30–50, the evidence will not be any different with a larger sample size [15]. Whilst this may be adequate for the process indicators, the small sample size may limit the external validity of the SSI rate.

The microbial testing limited the association between the SSI and type of microorganisms. Thus, potential studies should conduct adequate sampling for microbial testing and SSI association.

## 8 Recommendations for Clinical Practice

Lee et al. proposed that *the demonstrable power of surveillance is in sharing the findings with those who need to know and are able to act on patient safety* [37]. Consequently, feedback may prompt investigation into higher reported SSI rates than the benchmark and implement appropriate preventative measures to decrease the SSI rates [34]. Accordingly, the third objective of the project was to provide the MFOS unit with feedback as an opportunity to raise awareness and improve performance.

The Australian Commission on Safety and Quality in Health Care (ACSQHC) proposed that reporting on SSI rates should not be limited to clinicians but should also include reporting to the public in an effort to demonstrate quality improvement, promote public trust as well as support patient's choice [21]. This supports the suggestion that public engagement is necessary in the prevention of SSI [74]. Although, Kuster et al. cautions that public reporting may not be well received in health systems or environments where there are disincentives for unfavourable outcomes [70]. Thus, the findings from the current project, will be used to raise public/community awareness on the prevention of SSI. For example, the pre‐operative bathing and wound management post‐discharge from the health facility.

In addition, recommendations were made to the MFOS unit to formulate and adopt an evidence‐based pre‐operative SSI risk assessment algorithm for clinical practice; SAP guideline; SSI preventative strategies that are applicable to MFOS procedures, for example, *bundles of care* and wound management guideline.

## 9 Conclusion

SSI is an infection that occurs after surgery irrespective of the site of the surgical incision(s) [1]. The severity of SSIs varies from superficial skin infection to life threatening septicaemia [2]. Consequently, they are associated with significant morbidity and mortality of the affected patients with significant number of deaths occurring within 30‐days of the surgery [3].

The rationale for the clinical audit was to assess the prevention of SSI in clinical practice within the MFOS unit using the NICE clinical guideline (NG125) as the benchmark standard(s). The results indicated that there were lapses in infection control practices with subsequent low compliance rate. It is also acknowledged that there were process indicators, which were not necessarily applicable to all participants and may have contributed to low compliance. Of significance within the SA context was the lack of established SSISP as well as established guidelines on the prevention and management of SSI. Additionally, the results also showed that there was lack of a structured approach to the management of surgical incisional wounds.

The clinical impact of the SSI was indicated by the high SSI rate relative to previous studies in SA and globally. In this cohort, the SSI resulted in flap failure, sepsis and death. Thus, the necessity to implement preventative strategies to ameliorate the impact of SSI. To this end, feedback to the MFOS unit was provided to achieve this objective. Thereafter, evidence‐based clinical guidelines will be formulated for the MFOS unit, which can be translated into clinical practice though seminars, print media or other educational materials. This will facilitate acceptability and endorsement by clinicians. Despite several limitations such as small sample size and limited microbial testing, the clinical audit was successful in achieving the aim and objectives of the clinical audit. At a national level, it is recommended that the National Department of Health should establish a national surveillance programme with mandatory surveillance at a local level. Furthermore, guidelines on the prevention and management of SSI including wound care management should be established.

## Conflicts of Interest

The authors declare no conflicts of interest.

## Supporting information


Data S1.

Data S2.

Data S3.

## Data Availability

The data that support the findings of this study are available from the corresponding author upon reasonable request.
