# Supplementary material for: A Clinical Audit of Surgical Site Infection Surveillance in a Maxillo‐Facial and Oral Surgery Unit in an Academic Hospital Complex in South Africa
Source: Int Wound J. 2025 Apr 27;22(5):e70196. doi: 10.1111/iwj.70196 (PMC12034848; doi:10.1111/iwj.70196)
Supplement: Supplementary file 1 — Data S1. [file IWJ-22-e70196-s001.docx]

| Electronic version available from: <https://www.nice.org.uk/guidance/ng125/resources>  **CLINICAL AUDIT: PROCESS INDICATORS** | | Yes/No |
| --- | --- | --- |
| SSI 1 | Advise patients to shower or have a bath (or help patients to shower, bath or bed bath) using soap, either the day before, or on the day of, surgery. |  |
| SSI 2 | "Consider nasal mupirocin in combination with a chlorhexidine body wash before procedures in which Staphylococcus aureus is a likely cause of a surgical site infection. This should be locally determined and take into account:  • the type of procedure  • individual patient risk factors  • the increased risk of side effects in preterm infants (see recommendation 1.3.8)  • the potential impact of infection. " |  |
| SSI 3 | Do not use hair removal routinely to reduce the risk of surgical site infection. |  |
| SSI 4 | If hair has to be removed, use electric clippers with a single-use head on the day of surgery. Do not use razors for hair removal, because they increase the risk of surgical site infection. |  |
| SSI 5 | The operating team should remove hand jewellery before operations. |  |
| SSI 6 | The operating team should remove artificial nails and nail polish before operations. |  |
| SSI 7 | "Give antibiotic prophylaxis to patients before:  • clean surgery involving the placement of a prosthesis or implant  • clean-contaminated surgery  • contaminated surgery. " |  |
| SSI 8 | Administer surgical antibiotic prophylaxis in the 120 mins preceding surgical incision |  |
| SSI 9 | Give antibiotic treatment (in addition to prophylaxis) to patients having surgery on a dirty or infected wound. |  |
| SSI 10 | "The operating team should wash their hands prior to the first operation on the list using an aqueous antiseptic surgical solution, with a single-use brush or pick for the nails, and ensure that hands and nails are visibly clean.  surgical hand preparation be performed either by scrubbing with a  suitable antimicrobial soap and water or using a suitable ABHR before donning sterile gloves." |  |
| SSI 11 | Before subsequent operations, hands should be washed using either an alcoholic hand rub or an antiseptic surgical solution. If hands are soiled then they should be washed again with an antiseptic surgical solution. |  |
| SSI 12 | Prepare the skin at the surgical site immediately before incision using an antiseptic preparation. |  |
| SSI 13 | Be aware of the risks of using skin antiseptics in babies, in particular the risk of severe chemical injuries with the use of chlorhexidine (both alcohol-based and aqueous solutions) in preterm babies. |  |
| SSI 14 | Do not use diathermy for surgical incision to reduce the risk of surgical site infection. |  |
| SSI 15 | Maintaining normal body temperature (normothermia) (˃ 36° C) |  |
| SSI 16 | "If patient’s core temperature peri-operatively was at or below 36 degrees Celsius, were warming procedures used e.g. forced-air blankets, warmed IV fluids, warming blanket under patient on operation table, hats and booties?" |  |
| SSI 17 | Use of protocols for intensive perioperative blood glucose control |  |
| SSI 18 | Were serum glucose levels below 11.1mmol/L on the first 2 days post-operation? |  |
| SSI 19 | Was a glucose control protocol used- sliding scale or insulin IV? |  |
| SSI 20 | "Consider the use of irrigation of the incisional wound with an aqueous PVP-I solution before closure for the purpose of preventing SSI, particularly in clean and clean-contaminated wounds." |  |
| SSI 21 | When using sutures, consider using antimicrobial triclosan-coated sutures, especially for paediatric surgery, to reduce the risk of surgical site infection. |  |
| SSI 22 | Cover surgical incisions with an appropriate interactive dressing at the end of the operation. |  |
| SSI 23 | Use an aseptic non-touch technique for changing or removing surgical wound dressings. |  |
| SSI 24 | Do not use topical antimicrobial agents for surgical wounds that are healing by primary intention to reduce the risk of surgical site infection. |  |
| SSI 25 | "Do not use prolongation of SAP administration after completion of the  operation for the purpose of preventing SSI." |  |
| SSI 26 | Do not use Eusol and gauze, or moist cotton gauze or mercuric antiseptic solutions to manage surgical wounds that are healing by secondary intention. |  |
| SSI 27 | Use an appropriate interactive dressing to manage surgical wounds that are healing by secondary intention. |  |
| SSI 28 | Ask a tissue viability nurse (or another healthcare professional with tissue viability expertise) for advice on appropriate dressings for the management of surgical wounds that are healing by secondary intention. |  |
| SSI 29 | When surgical site infection is suspected by the presence of cellulitis, either by a new infection or an infection caused by treatment failure, give the patient an antibiotic that covers the likely causative organisms. Consider local resistance patterns and the results of microbiological tests in choosing an antibiotic. For information on antimicrobial stewardship programmes see the NICE guideline on antimicrobial stewardship: systems and processes for effective antimicrobial medicine use. |  |
| SSI 30 | Use a structured approach to care to improve overall management of surgical wounds. This should include preoperative assessments to identify people with potential wound healing problems. Enhanced education of healthcare workers, patients and carers, and sharing of clinical expertise is needed to support this. |  |
