# Supplementary material for: A Clinical Audit of Surgical Site Infection Surveillance in a Maxillo‐Facial and Oral Surgery Unit in an Academic Hospital Complex in South Africa
Source: Int Wound J. 2025 Apr 27;22(5):e70196. doi: 10.1111/iwj.70196 (PMC12034848; doi:10.1111/iwj.70196)
Supplement: Supplementary file 2 — Data S2. [file IWJ-22-e70196-s003.pdf]

Surgical site infection surveillance post-operative data collection form

World Health Organization

ID	Patient name	Age/ Date of birth ...../...../..........	InPatient number	Address (town/village)
	Telephone number 1	Whose telephone number		Checked? ☐
	Telephone number 2	Whose telephone number		Checked? ☐

All follow-up in the 30-day post-operative period should be recorded in ***Box 2****. Each patient interaction should be recorded in the "Event" column from the day of surgery onwards, including: surgical procedure, wound dressing removed/changed, (each) inpatient (IP) review, discharge, outpatient (OP) review, telephone call, readmission, return to the operating theatre, surveillance discontinued (reason). At least three reviews are recommended in the 30-day follow-up period. For each "Event", please record the date, tick the "Antibx" column if antibiotics are prescribed/being taken, complete health workers' initials, and record any surgical site infection (SSI) symptoms or other important notes in the last column (see footnote 1).*

BOX 2 - Admission date to hospital for primary operation: ..../......./.............. Hospital discharge date: ....../......./..............

Day	Date	Event	Antibx	SSI symptoms and other notes[1]	Health worker initials
1		Surgical procedure			
2-3					
4-5					
6-7					
8-10					
11-14					
15-17					
18-21					
22-25					
26-29					
Day 30				End of SSI surveillance (standard)	

1. *At each patient interaction, first check the patient's identification. Then assess or ask about the* ***SSI symptoms****:*
- *Drainage of fluid from wound: pus versus clear (serous) / bloody / other • Pain / tenderness beyond normal for operation*
- *Localized swelling or wound breakdown • Redness/heat of skin • Generally unwell, especially fever >38°C*

If any SSI symptoms are noted in Box 2, proceed to Box 3 to determine the SSI case definition and consult with the operating surgeon.

BOX 3

Surgical Site Infection? ☐ Yes ☐ No (Determine with case definition tick boxes below)

Patient re-admitted for Surgical Site Infection? ☐ Yes ☐ No (note reason) ....

Date of re-admission for Surgical Site Infection: ...../...../.......... Discharge date**:** ...../...../..........

☐ **Superficial SSI** (skin/subcutaneous) e.g. cellulitis

☐ Purulent drainage (pus) from superficial incision

OR

☐ Organism identified (if culture done)*

OR

☐ Superficial incision deliberately re-opened

AND

☐ Infection symptoms[1]

OR

☐ Surgeon/attending physician diagnosis

☐ **Deep SSI** (fascia/muscle) e.g. deep abscess

☐ Purulent drainage (pus) from deep incision

OR

☐ Deep incision dehiscence or deliberately opened by surgeon

AND

☐ Organism identified (if culture done)*

AND

☐ Infection symptoms[1]

OR

☐ Deep infection/abscess found on imaging/examination

☐ **Organ/space SSI**** Deeper than fascia/muscle e.g. endometritis (organ), peritonitis (space)

☐ Purulent drainage (pus) from sterile organ or space (from an inserted drain)

OR

☐ Organ or space infection/abscess found on imaging/examination

OR

☐ Organism identified from fluid/tissue from organ/ space*

Other surgical complications

☐ Non-infectious **local** wound complications including bleeding and abnormal skin reactions

☐ Patient death: Date ...../...../.......... Cause of death (as far as known) ....

Microbiology culture results*	Specimen taken	Organism(s) identifed	Antibiotic resistance/sensitivities
	Date...../...../.......... type.............		

*Note: most surgical wounds that have broken down rapidly become colonized with bacteria. Bacterial growth from a wound is only significant when a sample to identify organisms by microbiological culture is collected aseptically under sterile conditions with symptoms of infection also present.

Date form completed ..../......../............... Database entry [Y / N] Signature..

Key explanations to complete the post-operative form

Whose phone number = patient (mobile or home), or family member, or neighbour, or friend

Checked = phone number called to check before patient leaves hospital

****List of specific organ/space infection sites**

Code	Site	Code	Site
BONE	Osteomyelitis	MED	Mediastinitis
BRST	Breast abscess or mastitis	MEN	Meningitis or venticulitis
CARD	Myocarditis or pericarditis	ORAL	Oral cavity (mouth, tongue, or gums)
DISC	Disc space	OREP	Other infections of the male or female reproductive tract
EAR	Ear, mastoid	PJI	Periprosthetic joint infection
EMET	Endometritis	SA	Spinal abscess without menigitis
ENDO	Endocarditis	SINU	Sinusitis
GIT	Gastrointestinal tract	UR	Upper respiratory tract
IAB	Intraabdominal, not specified	USI	Urinary System infection
IC	Intracranial, brain abscess or dura	VASC	Arterial or venous infection
JNT	Joint or Bursa	VCUF	Vaginal cuff
LUNG	Other infections of the lower respiratory tract		

To understand specific criteria for defining these infections please refer to CDC/NHSN Surveillance Definitions for Specific Types of Infections https://www.cdc.gov/nhsn/PDFs/pscManual/17pscNosInfDef_current.pdf
