# Supplementary material for: A Clinical Audit of Surgical Site Infection Surveillance in a Maxillo‐Facial and Oral Surgery Unit in an Academic Hospital Complex in South Africa
Source: Int Wound J. 2025 Apr 27;22(5):e70196. doi: 10.1111/iwj.70196 (PMC12034848; doi:10.1111/iwj.70196)
Supplement: Supplementary file 3 — Data S3. [file IWJ-22-e70196-s002.pdf]

Surgical site infection surveillance peri-operative data collection form

ID	Patient name	Age/ Date of birth ..../..../.........	InPatient number	Date of admission ..../..../.........
	Primary diagnosis	Sex ☐ F ☐ M	Surveillance number	

World Health Organization

1

Surgical procedure.....................................

Date of surgery...

Operating theater [ ]

Lead surgeon name..

Grade...

CDC – NNIS Risk Index Variables

2

ASA class

☐ 1. Normal healthy person
☐ 2. Mild systemic disease (e.g. hypertension, well controlled diabetes)
☐ 3. Severe systemic disease not incapacitating (e.g. moderate COPD, diabetes, malignancy)
☐ 4. Incapacitating systemic disease that is a constant threat to life (e.g. pre-eclampsia, heavy bleeding)
☐ 5. Moribund patient, not expected to survive with or without operation (e.g. major trauma)

Weight.................. kg
Height...................cm

3

Surgical wound class

Clean ☐ = Sterile tissue with no resident bacteria e.g. neurosurgery
Clean-contaminated ☐ = CONTROLLED entry to tissue with resident bacteria e.g. hysterectomy
Contaminated ☐ = UNCONTROLLED entry to tissue with bacteria e.g. acute gastrointestinal perforation
Dirty / infected ☐ = Heavy contamination (e.g. soil in wound) or infection already established

4

Start time (knife to skin)
[:] 24h clock
End time (skin closure)
[:] 24h clock
Duration =............hrs ....mins

Urgency of operation

☐ Emergency – must be done immediately to save life (e.g. major bleed)
☐ Urgent – must be done within 24-48h (e.g. repair of fracture)
☐ Semi-elective – must be done within days-weeks (e.g. tumour removal)
☐ Elective – no time constraints (e.g. cosmetic procedure)

PRE/PERI-OPERATIVE PROCESS MEASURES

5

Patient preparation

Pre-op bath / shower (full body [Y / N] Date ...../...../..........
Antimicrobial soap used [Y / N] Plain soap used [Y / N]
Hair removal (HR): ☐ Razor ☐ Clippers ☐ None
HR Date .... ☐ Home ☐ Ward ☐ Theatre

Surgical antibiotic prophylaxis

☐ No prophylaxis required
Required but not given due to: ☐ Unavailable
☐ Other ....

Antibiotic given:

☐ Co-amoxiclav ☐ Cefazolin ☐ Cloxacillin ☐ Vancomycin
☐ Ciprofloxacin ☐ Gentamicin ☐ Metronidazole ☐ Penicillin
☐ Other antibiotic......................... Dose....................... (mg)
Time given [:] 24h clock Time re-dosed [:] 24h clock

Postoperative antibiotics

Were antibiotics ceased at completion of surgery? [Y / N]
If not, what antibiotics were prescribed?
Drug...................................... Dose................................ (mg)
Doses / day............................ Duration (days) ....

Reason given

☐ Post-op prophylaxis ☐ Drain / implant inserted
☐ Treating suspected / known infection ☐ Other ....

Surgical skin preparation (under sterile conditions)

☐ Chlorhex-alc ☐ Iodine+alc ☐ Chlorhex-aq ☐ Iodine-aq
Appropriate skin preparation technique [Y / N]
Allowed to fully dry [Y / N]

Surgical hand preparation

☐ Alcohol-based hand rub ☐ Antimicrobial soap+water
☐ Plain soap+water
Time spent on procedure [ ] mins [ ] secs
Appropriate hand preparation technique: [Y / N]

Theatre traffic

Headcount at start of operation............................ total ......
Number of entries during operation.......................
Door openings during operation........................... total ......

Drain / implant

Location...
Drain inserted? [Y / N]
If YES, type of drain: ☐ Open ☐ Closed
Antibiotic given in presence of drain but no infection?
[Y / N]
Implant used? [Y / N]
☐ Metal (Ortho) ☐ Skin graft ☐ Mesh ☐ Other

6 **Other measure(s) – decided at local level**...

....

Date form completed ..../......../................ Database entry [Y / N] Signature...

Key explanations to complete the peri-operative form

Box 1

Surgical procedure - refers to an operation where at least one incision (including a laparoscopic approach) is made through the skin or mucous membrane, or reoperation via an incision that was left open during a prior operative procedure AND takes place in an operating theatre – select the exact surgical procedure from the list below.

Abdominal aortic aneurysm repair
Limb amputation
Appendix surgery
Shunt for dialysis
Bile duct, liver or pancreas surgery
Breast surgery
Cardiac surgery
Carotid endarterectomy
Coronary artery bypass surgery – donor + graft sites
Coronary artery bypass surgery – chest only
Gallbladder surgery
Colon surgery
Craniotomy
Caesarean section
Spinal fusion
Open reduction of fracture
Gastric surgery
Herniorrhaphy
Hip prosthesis
Heart transplant
Abdominal hysterectomy
Knee prosthesis
Kidney transplant
Laminectomy
Liver transplant
Neck surgery
Kidney surgery
Ovarian surgery
Pacemaker surgery
Prostate surgery
Peripheral vascular bypass surgery
Rectal surgery
Refusion of spine
Small bowel surgery
Spleen surgery
Thoracic surgery
Thyroid and/or parathyroid surgery
Vaginal hysterectomy
Ventricular shunt
Abdominal surgery

Grade of surgeon - senior (surgeon with more than 10 years of experience in total); junior (surgeon with less than 10 years of experience); trainee (junior doctor who is in training in the surgical specialty); 'other grade' of surgeon (as defined locally).

Box 3

Surgical wound class -

1. Clean refers to an uninfected operative wound in which no inflammation is encountered and the respiratory, alimentary, genital or uninfected urinary tracts are not entered. In addition, clean wounds are primarily closed and, if necessary, drained with closed drainage. Operative incisional wounds that follow non-penetrating (blunt) trauma should be included in this category if they meet the criteria.

2. Clean-contaminated refers to operative wounds in which the respiratory, alimentary, genital or urinary tracts are entered under controlled conditions and without unusual contamination. Specifically, operations involving the biliary tract, appendix, vagina and oropharynx are included in this category, provided no evidence of infection or major break in technique is encountered.

3. Contaminated refers to open, fresh, accidental wounds. In addition, operations with major breaks in sterile technique (for example, open cardiac massage) or gross spillage from the gastrointestinal tract, and incisions in which acute, non-purulent inflammation is encountered, including necrotic tissue without evidence of purulent drainage (for example, dry gangrene), are included in this category.

4. Dirty or infected includes old traumatic wounds with retained devitalized tissue and those that involve existing clinical infection or perforated viscera. This definition suggests that the organisms causing postoperative infection were present in the operative field before the operation.

Box 5

Patient pre-operative bath/shower – patient shower or bath should be performed with either antimicrobial soap or plan soap, ideally 1-2 hours before the operation or at least the night before.

Appropriate surgical hand preparation (scrubbing) - an antiseptic (antimicrobial soap and water) handwash or antiseptic handrub (alcohol-based handrub product classified as high quality), performed **immediately** preoperatively to eliminate transient flora and reduce resident skin flora (such antiseptics often have persistent antimicrobial activity). The technique should be the WHO recommended steps, including drying. Length of time is according to the manufacturers' instructions, typically 2-5 minutes for soap and water; for alcohol–based handrub follow manufacturers' instructions (*http://www.who.int/gpsc/5may/hh-surgicalA3.pdf?ua=1*).

Appropriate surgical skin preparation (under sterile conditions) – use of sterile gauze/sponge and instruments, with movements from clean to dirty areas, that is, from the centre of the incision site outwards, maintaining aseptic technique and covering a broad area of the patient's skin, to be performed immediately before draping and incision. No areas touched that are not part of the preparation area. Allow to fully dry before incision.
